# Abnormal Visual Evoked Responses to Emotional Cues Correspond to Diagnosis and Disease Severity in Fibromyalgia

**DOI:** 10.3389/fnbeh.2022.852133

**Published:** 2022-05-04

**Authors:** Noam Goldway, Nathan M. Petro, Jacob Ablin, Andreas Keil, Eti Ben Simon, Yoav Zamir, Libat Weizman, Ayam Greental, Talma Hendler, Haggai Sharon

**Affiliations:** ^1^Sagol Brain Institute, Wohl Institute for Advanced Imaging, Tel-Aviv Sourasky Medical Centre, Tel Aviv-Yafo, Israel; ^2^Sagol School of Neuroscience, Tel Aviv University, Tel Aviv-Yafo, Israel; ^3^Department of Psychology, New York University, New York, NY, United States; ^4^Institute for Human Neuroscience, Boys Town National Research Hospital, Boys Town, NE, United States; ^5^Sackler School of Medicine, Tel Aviv University, Tel Aviv-Yafo, Israel; ^6^Department of Internal Medicine, Tel Aviv Sourasky Medical Centre, Tel Aviv-Yafo, Israel; ^7^Department of Psychology, Center for the Study of Emotion and Attention, University of Florida, Gainesville, FL, United States; ^8^Department of Psychology, University of California, Berkeley, Berkeley, CA, United States; ^9^School of Psychological Sciences, Tel Aviv University, Tel Aviv-Yafo, Israel; ^10^Department of Anesthesiology and Critical Care Medicine, Institute of Pain Medicine, Tel Aviv Sourasky Medical Centre, Tel Aviv-Yafo, Israel

**Keywords:** chronic pain & fibromyalgia, ssVEP (steady-state visual evoked potential), attention bias dynamics, emotion regulation, EEG

## Abstract

**Background:**

Chronic pain disorders are often associated with cognitive-emotional dysregulation. However, the relations between such dysregulation, underlying brain processes, and clinical symptom constellations, remain unclear. Here, we aimed to characterize the abnormalities in cognitive-emotional processing involved in fibromyalgia syndrome (FMS) and their relation to disease severity.

**Methods:**

Fifty-eight participants, 39 FMS patients (35F), and 19 healthy control subjects (16F) performed an EEG-based paradigm assessing attention allocation by extracting steady-state visually evoked potentials (ssVEP) in response to affective distractors presented during a cognitive task. Patients were also evaluated for pain severity, sleep quality, depression, and anxiety.

**Results:**

EEG ssVEP measurement indicated that, compared to healthy controls, FMS patients displayed impaired affective discrimination, and sustained attention to negative distractors. Moreover, patients displayed decreased task-related fronto-occipital EEG connectivity. Lack of adaptive attentional discrimination, measured via EEG, was predictive of pain severity, while impairments in fronto-occipital connectivity were predictive of impaired sleep.

**Conclusions:**

FMS patients display maladaptive affective attention modulation, which predicts disease symptoms. These findings support the centrality of cognitive-emotional dysregulation in the pathophysiology of chronic pain.

## Introduction

Pain is a complex perceptual phenomenon that involves sensory as well as affective and attentional aspects (Wiech, 2016). Attentional processes have a modulatory effect on acute pain perception (Villemure and Bushnell, 2002) and a key role in the transition from acute to chronic pain (Baliki et al., 2006, 2011, 2012; Bushnell et al., 2013; Baliki and Apkarian, 2015). When pain becomes chronic, impairments in attentional and affective control mechanisms are accompanied by depressive and anxious symptoms, as well maladaptive sleep (Korff and Simon, 1996; Means-Christensen et al., 2008; Choy, 2015). This leads to additional worsening of the clinical status, with ongoing pain increasing the severity of affective symptoms, which, in turn, amplify chronic pain in a vicious cycle (Pincus and Williams, 1999; Koechlin et al., 2018). The relations between chronic pain and affective disorders were suggested to be mediated by attentional and cognitive biases (Pincus and Morley, 2001). However, the attentional mechanisms by which these relations are mediated have yet been explored.

In this study, we examined the contribution of affect-biased attention, i.e., the predisposition to attend to affective stimuli (Todd et al., 2012), to the manifestations of chronic pain and affective symptomatology. We examined these relations in patients suffering from fibromyalgia syndrome (FMS), a disorder in which distributed chronic pain is accompanied by abnormalities in emotion regulation, depressive and anxious symptomatology, and maladaptive sleep patterns (Häuser et al., 2015).

**FIGURE 1:**
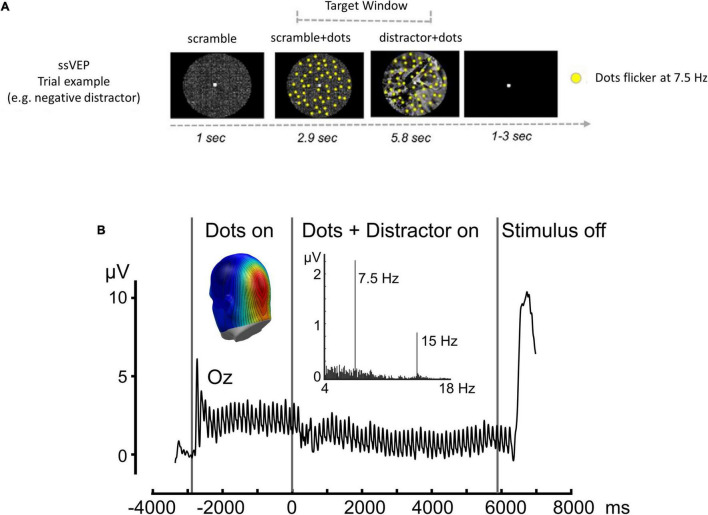
ssVEP task. **(A)** Time sequence for a single trial: intervals of coherent motion could occur between 1.17 and 7 s post-stimulus onset (target window). Each trial lasted 9.751 s with a variable 3–5-s inter-trial interval. **(B)** Time-domain representation of the steady-state visual evoked potentials, when viewing dots flickering at a rate of 7.5 Hz, recorded from sensor Oz. Black vertical lines indicate the duration of the stimulus presentation. Insets display the frequency spectrum of the same data, with a pronounced peak at the flickering frequency (7.5 Hz) and the first harmonic frequency (15 Hz) clearly visible. The inset shows a back view of the spectral amplitude topography of this response as projected to a standard head. Note the focal parieto-occipital distribution of the steady-state visual evoked potential signal evoked by flickering dots. Adapted with permission from Simon et al. (2015).

To assess affect-biased attention, we continuously recorded EEG signals while participants were required to detect minor changes in the movements of task stimuli (flickering dots) that were superimposed on distracting background pictures of either aversive or neutral content (Figure 1A). We used steady-state visually evoked potentials (ssVEPs), a measure of cortical response to a flickering stimulus that is oscillating at a known frequency (Müller et al., 1997), as our signal of interest. Based on previous studies that observed modulation of the ssVEP signal as a function of instructed attention (Müller et al., 2003), fear conditioning (Miskovic and Keil, 2013; McTeague et al., 2015), and emotional arousal (Keil et al., 2003, 2012), ssVEP amplitude was used to evaluate the level of distraction induced by the background pictures: smaller task-related amplitudes indicate a greater level of distraction from the task stimulus (Figure 1B). Previous investigations that applied a similar approach demonstrated an alternation in the modulation of the ssVEP signal in sleep-deprived (Simon et al., 2015), depressed (Moratti et al., 2008) and anxious individuals (Wieser et al., 2011, 2012; McTeague et al., 2018).

Our first aim was to characterize patterns of affect-biased attention in the presence of chronic pain. According to previous findings, FMS patients display reduced ability to discriminate between threatening and non-threatening sensory signals (Meulders et al., 2017, 2018) as well as sustained attention to negative cues (Duschek et al., 2014). We therefore predicted that negative attentional bias in FMS will manifest as initial attentional bias toward neutral stimuli, representing an avoidance pattern, followed by sustained attention to negative stimuli after a period of habituation. Moreover, we hypothesized that impaired attentional modulation in FMS will correspond to abnormal top-down control (Montoya et al., 2005; Choy, 2015). To this end, we also evaluated task-related fronto-occipital EEG connectivity, an index that was shown to correspond with fMRI functional connectivity between the pre-frontal cortex and amygdala (Simon et al., 2015). Finally, we examined correlations between these EEG indices and clinical disease attributes: Pain, depression, anxiety, and sleep impairments.

## Materials and Methods

### Participants

58 participants took part in a between-group design (ClinicalTrials.gov Identifier: NCT02146495): 39 FMS patients (19–64 years old, mean, 36.67 ± 12.5 years; 35 females) and 19 healthy controls (HC, 23–56 years old, mean, 31.5 ± 9.2 years; 16 females). Patients were recruited from the fibromyalgia clinic of the Institute of Rheumatology and the Institute of Pain Medicine at the Tel Aviv Sourasky Medical Center in Israel. All patients had a diagnosis of fibromyalgia according to the ACR, 2010 criteria (Wolfe et al., 2011) which was confirmed by a clinical interview and physical examination by a board-certified rheumatologist or a pain specialist. Exclusion criteria included other diagnosed chronic pain syndromes, major neuropsychiatric illness, and changes in pharmacotherapy during the 2 months preceding recruitment, or an intended upcoming change in the treatment. In addition, 19 healthy volunteers participated in the study. Participants in the control group did not suffer from sleep, neurologic, or psychiatric disorders (assessed using a detailed medical history questionnaire and clinical interview), and were not taking any chronic medications. The study was approved by the Tel-Aviv Sourasky Medical Center institutional review board, and all participants provided written informed consent.

### Clinical Evaluation

The health status of the control group was verified using a detailed medical history questionnaire that verified the lack of existing neurologic or psychiatric disorders. Clinical manifestations of FMS (i.e., pain, sleep experience, anxiety, and depression) were evaluated using self-report questionnaires: the McGill pain questionnaire (Melzack, 1975), the Pittsburgh sleep quality index (PSQI; Buysse et al., 1989), the Trait Anxiety Inventory (STAI-T; Gaudry et al., 1975) and the Beck’s Depression Inventory (BDI; Beck et al., 1961). The relations between FMS severity and ssVEP indices were assessed using Spearman correlations.

### Stimulus and Procedure

ssVEPs were evoked using randomly moving dots flickering at a rate of 7.5 Hz, while superimposed on emotional or neutral background pictures (as previously described by Ben-Simon et al., 2015). In short, participants were instructed to focus their attention on the randomly moving flickering dots and detect very short intervals of coherently moving dots while ignoring the presented distractors (Figure 1). Thus, the magnitude of the ssVEP amplitude was used as a proxy for the level of distraction caused by background affective distractors. A full description of the ssVEP paradigm can be found in the Supplementary Material.

### EEG Recording

EEG data were acquired using the V-Amp™ EEG amplifier (Brain Products™, Munich Germany) and the BrainCap™ electrode cap with sintered Ag/AgCI ring electrodes providing 16 EEG channels (Fc1, Fc2, Fz, F3, F4, F7, F8, Fp1, Fp2, Cz, Pz, P7, P8, Oz, O1, O2) and two ECG channels. (Falk Minow Services™, Herrsching-Breitburnn, Germany). Electrodes were positioned according to the standard 10/20 system. The reference electrode was between Fz and Cz. The raw EEG signal was sampled at 250 Hz and recorded using the Brain Vision Recorder™ software (Brain Products) with scalp impedance for each electrode kept below 20 kΩ.

### ssVEP Analysis

Time-varying amplitudes at the stimulation frequency (7.5 Hz) were extracted employing a Hilbert transformation of the time-domain averaged data using in-house MATLAB scripts: data were filtered with a 10th-order Butterworth bandpass filter having a width of 0.5 Hz around the center frequency of 7.5. The time-varying amplitude was extracted as the complex conjugate of the bandpass-filtered signal and the Hilbert-transformed analytic signal for each time point. The temporal smearing introduced by this procedure was 440 ms as measured by the full width at half maximum of the impulse response function. Subsequently, mean amplitudes were extracted across occipital electrodes (O1, O2, and Oz), at a time window corresponding to distractor onset (between 3600 and 3700 ms) and subtracted from the mean amplitudes of the baseline segment (the presentation of the scrambled picture, 2000–3000 ms). Based on previous studies that investigated the ssVEP dynamics in response to emotional content (Deweese et al., 2014), we have divided the ssVEP time-series into early (0–900 ms after stimulus onset) and late (900–3000 ms after stimulus onset) time windows (see also Supplementary Material). Mean values were extracted to SPSS version 20 and analyzed using an analysis of variance (ANOVA) examining the effect of three factors: (1) distractor valence (negative/neutral), (2) Group (FMS/HC), and (3) time window of stimulus display (early/late). The main effects and interactions were tested using a significance of *p* < 0.05 and 90% CI was calculated for the effect size η2. *P* values in *post hoc* analyses were corrected using the conservative Scheffé’s procedure, correcting for all possible contrasts (Scheffé, 1953).

In addition, we extracted the time-varying inter-electrode phase-locking value, calculated as the stability of the complex phase across trials, relative to the site Oz (Lachaux et al., 1999). Specifically, we used the real and imaginary part of the Hilbert transform calculated for each trial, time point, and electrode to yield an index of time-varying complex phase. In order to eliminate amplitude bias, these instantaneous phase values were normalized by dividing them by their absolute values. Subsequently, for each time point, the complex phase at electrode Oz was subtracted from all other electrodes, resulting in an electrodeXtime point matrix of complex difference values. These values were again normalized to be on a unit circle (divided by their amplitude) and then averaged across the trials of a given experimental condition (for a similar procedure, see Keil et al., 2007). The resulting inter-phase-locking indices (iPLI) are bounded between 0 and 1, and can be interpreted to reflect the similarity of the phase difference between two electrodes across trials, at a given moment in time, with 1 indicating full identity of the phase difference and 0 indicating complete statistical independence. To allow mapping and to avoid distortions of the scale by the perfect phase locking of Oz with itself, the values at Oz were replaced by a weighted mean of the spline interpolated values of the remaining electrodes. However, these values did not enter the statistical analysis. To examine changes in frontal connectivity patterns, Fp1 and Fp2 phase-locking relative to the site Oz indices were analyzed using the three-way ANOVA model that was used for the ssVEP analysis.

## Results

### FMS Clinical Characteristics

The mean score of the McGill pain questionnaire was 24.8 (± 10.5 SD) [no standard clinical cutoff (Melzack and Katz, 2001). These results are similar to previous investigations that used this questionnaire with FMS patients (e.g., Perry et al., 1988; Burckhardt et al., 1992)]. The mean anxiety rating assessed using STAI-T was 49.1 (± 11) [20–37: no or low anxiety, 38–44: moderate anxiety, and 45–80: high anxiety (Knight et al., 1983; Kayikcioglu et al., 2017)]. Depression scores from the BDI questionnaire had a mean score of 19.1 (± 9.3) [14–19: mild depression, 20–28: moderate depression, 29–63: severe depression (Beck et al., 1996)] and sleep quality assessed using the PSQI was 12.1 (± 4.1) [clinical cutoff: 5 (Buysse et al., 1989, 2008)]. Notably, pain ratings were correlated with anxiety [R_Spearman_ = 0.39, 95% CI [0.08;0.63], *p* = 0.016] and depression [R_Spearman_ = 0.46, 95% CI [0.17;0.68], *p* = 0.004] scores, but not with sleep quality scores [R_Spearman_ = 0.17, 95%CI [-0.17;0.47], *p* = 0.3]. Affective symptoms (depression and anxiety) were highly correlated [R_Spearman_ = 0.78, 95% CI [0.61;0.88], *p* < 0.001] and sleep quality correlated with depression [R_Spearman_ = 0.34, 95% CI [0.13;0.6], *p* < 0.04] but not with anxiety [R_Spearman_ = 0.27, 95% CI [−0.06;0.55], *p* = 0.12] (see Supplementary Figure 1). Self-report data were missing for two FMS patients. One additional patient did not fill out the PSQI questionnaire.

### ssVEP Response

**FIGURE 2:**
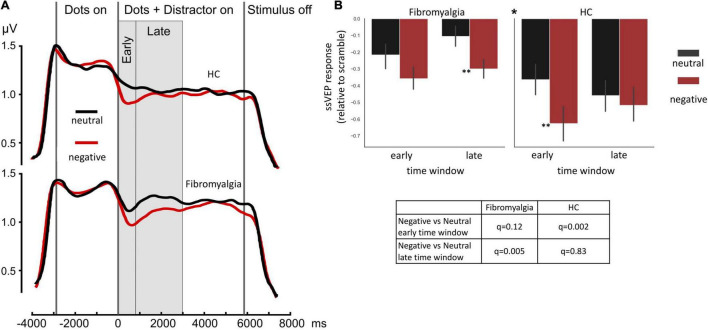
ssVEP amplitudes. **(A)** Mean group results of a time sequence of a single trial in the ssVEP task. Each trial started with a 1-second presentation of a scrambled picture, followed by the appearance of flickering dots (at a rate of 7.5 Hz) for 2.9 s. Consequently, a negative or neutral picture appeared for 5.8 s behind the dots. **(B)** Bar graphs illustrate the mean of ssVEP amplitude change relative to baseline in the early and late time windows, indicating different patterns of response, specifically a triple interaction of group*time-window*distractor-type driven by a large difference between distractor types at the early time window in the healthy control group (HC panel, left-hand set of bars), but not in the FMS group (Fibromyalgia panel, left-hand set of bars) and stronger response to negative compare the neutral stimulus in the FMS (Fibromyalgia panel, right-hand set of bars) but not in the HC group (HC panel, right-hand set of bars) in the late time window. Error bars represent SEM. The * at the middle top part of panel **(B)** is indicating the significant triple interaction group*time-window*distractor-type. Significant *post hoc t*-tests for the differences between distractor types, within each time window within each group are also indicated. *p < 0.05 ^**^p < 0.005. *Post hoc* Scheffé correction. This analysis is based on data from 58 participants (39 FMS and 19HC).

Negative distractors induced a greater decrease in ssVEP amplitude compared with neutral distractors across all participants [*F*(1,56) = 18, η^2^ = 0.24, 90% CI [0.09;0.38], *p* < 0.001] (main effect for distractor valence). FMS patients displayed overall reduced responsivity to task stimulus [*F*(1,56) = 7, η^2^ = 0.11, 90% CI [0.01;0.24], *p* = 0.01] (main effect for group). Most importantly, the ANOVA model revealed a significant triple time-window*group*distractor-valence interaction [*F*(1,56) = 6.1, η^2^ = 0.1, 90% CI [0.01;0.23], *p* = 0.017], indicating different dynamics of the ssVEP response. Further exploring this interaction, *post hoc* tests revealed that during the early time window, HC subjects (but not FMS patients) were able to discriminate between neutral and negative distractors (PScheffe HC = 0.002, PScheffe FMS = 0.12). In the late time window, this pattern was flipped: FMS patients showed a strong attentional shift to the negative stimulus, while HC showed adaptation to this type of distractor (PScheffe HC = 0.83, PScheffe FMS = 0.005; Figure 2).

### EEG Connectivity

**FIGURE 3:**
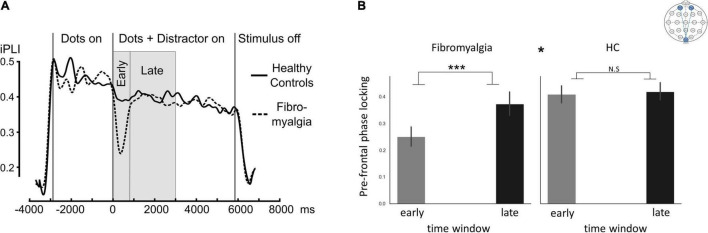
ssVEP connectivity. “Fronto-occipital connectivity,” indicating Oz phase-locking indices with frontal electrodes (Fp1 and Fp2). **(A)** Mean inter-site phase-locking index (iPLI) time sequence during a single trial in the ssVEP task across all distractor types. **(B)** Bar graphs describing the mean phase-locking index across distractor types in early and late time windows. The * at the middle top part of panel **(B)** is indicating the significant group*time window interaction. Significant *post hoc t*-tests for the differences between time windows within each group. Error bars represent SEM. *p < 0.05, ***p < 0.0005. *Post hoc* Scheffé correction. This analysis is based on data from 58 participants (39 FMS and 19HC).

Fronto-occipital phase-locking values presented different dynamics between patients and HC as indicated by Time-window*group interaction [*F*(1,56) = 7.8, η^2^ = 0.12, 90% CI [0.02;0.26], *p* = 0.007]. Follow-up analysis revealed time-dependent differences (early/late time-window) in the FMS (PScheffe > 0.001) but not in the HC group (*q* = 0.99; Figure 3).

### Correlation Between ssVEP Indices and Disease Symptoms

To examine our hypotheses that abnormal patterns detected in the EEG would have clinical significance, we examined the correlation between the three ssVEP indices that were altered in FMS patients and clinical symptomatology measured by the general scores of the McGill, STAI-T, BDI, and PSQI questionnaires. Three EEG indices showed a different pattern between the HC and FMS groups. The first was the difference between negative and neutral ssVEP amplitude in the *early* time window. Such a difference was evident only in the control group. This result replicates a similar pattern of ssVEP response shown in previous studies (e.g., Deweese et al., 2014; Simon et al., 2015), indicating that negative stimulus is more distracting than the neutral stimulus during the initial response. The lack of differential response in the early time window was a characteristic of the FMS group. The second component that was different between the groups was the difference between negative and neutral ssVEP amplitude in the late time window. Here, the between-group pattern was reversed: in HC, the negative distractor became less distracting over time, producing a distraction effect that is similar to the one caused by the neutral stimulus. In FMS patients, the differentiation between negative and neutral stimulus became apparent only during this late time widow, possibly reflecting sustained attention toward the negative stimulus. Finally, the third difference between the groups was fronto-parietal desynchronization observed during picture onset, which was evident only in the FMS group.

**FIGURE 4:**
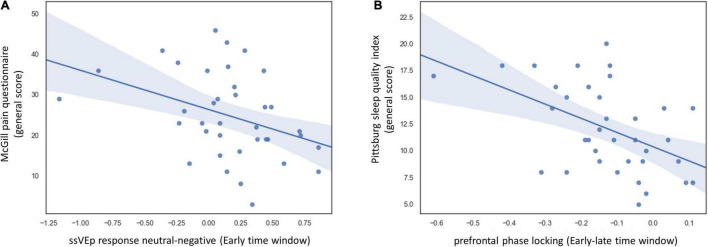
Correlation between task ssVEP indices and disease symptoms in the FMS group. **(A)** Scatter plot displaying the relation between and the general score of the McGill pain questionnaire and difference in ssVEP response between neutral and negative distractors during the early time window. To make sure that this result could not be attributed to affective symptom severity, we followed up on this analysis using partial correlation, controlling for depression, anxiety, and sleep quality scores. Controlling these variables did not diminish the correspondence between valance specific EEG response and pain severity [R_Spearman_ = 0.43, 95% CI [0.12:0.66], *p* = 0.01]. **(B)** Scatter plot displaying the relation between overall sleep quality as indicated by the Pittsburg sleep quality index and the delta between fronto-occipital phase-locking values in the early and late time windows. When controlling for additional clinical variables (anxiety, depression, and pain ratings) the correlation became stronger [R_Spearman_ = 0.57, 95% CI [0.3:0.76], *p* = 0.001]. Self-report data were missing for two FMS patients. One additional patient did not fill out the PSQI questionnaire. The analysis in panel **(A)** is based on data from 37 FMS patients and in panel **(B)**. Data from 36 FMS patients.

We correlated these EEG indices with the four main scales measuring symptom severity: the general scores of the McGill, STAI-T, BDI, and PSQI questionnaires. To correct for multiple comparisons, we used the FDR method (Benjamini and Hochberg, 1995). This analysis revealed that initial valance-specific ssVEP response (the difference between neutral and negative stimulus in the early time-window) was correlated with patients’ pain severity assessed by the McGill questionnaire [R_Spearman_ = 0.42, 95% CI [0.11:0.66], *q* = 0.05] (Figure 4A). To make sure that this result could not be attributed to affective symptoms severity, we applied partial correlation calculation, controlling for depression, anxiety, and sleep quality scores. Controlling for these variables did not diminish the correspondence between valance specific EEG response and pain severity [R_Spearman_ = 0.43, 95% CI [0.12:0.66], *p* = 0.01]. Additionally, the difference in phase-locking values (iPLI) between early and late time-windows, indicating transient disruption of fronto-occipital synchrony upon viewing the distractor picture, corresponded to subjective sleep disturbances (PSQI score) [R_Spearman_ = 0.49, 95% CI [0.19:0.71], *q* = 0.03] (Figure 4B). When controlling for additional clinical variables (anxiety, depression, and pain ratings) the correlation became stronger [R_Spearman_ = 0.57, 95% CI [0.3:0.76], *p* = 0.001]. Subjective anxiety (STAI-T), as well as the severity of depressive symptoms (BDI), was not correlated with any ssVEP indices (all *p* > 0.39).

## Discussion

The current study aimed to characterize the relations between abnormalities in neural processing of affect-biased attention, and somatic and affective symptoms of chronic pain. Our results regarding EEG dynamics revealed that FMS patients display altered attention toward negative emotional content compared to controls in primary visual areas as well as in higher-level modulatory circuits. Relative to controls, FMS patients displayed initially impaired discrimination between neutral and negative emotional stimuli, which was followed by sustained attention toward the negative stimuli, suggesting a reduction in normal habituation.

Loss of context-dependent emotional discrimination was predictive of pain severity. Specifically, increased initial attention to the neutral relative to negative stimuli was correlated with more severe clinical pain. This is in line with the generalization pattern seen in FMS in which neutral somatic information is interpreted as aversive (Häuser et al., 2015). Interestingly, a similar pattern of results was recently observed in a study of patients with social anxiety using a similar ssVEP paradigm (McTeague et al., 2018). In this study, the authors reported that while in most patients increased attention to negative stimuli corresponded to disease severity, patients with extreme anxiety displayed a different pattern, wherein neutral content was more distracting than negative. The authors speculated that in the presence of severe anxiety there is an initial exaggerated avoidance pattern. Our results resonate with this finding, as in our patient group increased attention during initial response to the neutral rather than to the negative stimulus, as measured using the ssVEP signal, was correlated with chronic pain severity. In addition, the fact that EEG markers of attentional-affective processing corresponded to somatic rather than affective symptomatology, is in line with the view that alterations in attention allocation play a pivotal role in the transformation from adaptive pain sensations to a chronic subjective experience of suffering. It also resonates with other results from studies conducted with FMS population that found connections between attentional biases and pain severity, but not affective symptomatology (Duschek et al., 2014). Our observations underscore the fact that pain, similar to other affective percepts, is constructed by individual tendencies and biases, that together determine the subjective quality (qualia) of our experience (Baliki and Apkarian, 2015). As biases become more ingrained, even a stimulus that does not normally carry aversive value may produce a negative experience.

In our FMS cohort, decreased fronto-occipital phase-locking values corresponded to the severity of sleep disturbance. In accordance, it was previously shown that sleep deprivation, produces similar abnormalities in EEG connectivity to those found here, and further corresponded to impaired frontal control on limbic regions (Simon et al., 2015). Notably, abnormal prefrontal-limbic-sensory connectivity is thought to give rise to attention biases to irrelevant threats, including difficulty to disengage and avoidance (Okon-Singer, 2018). The role of impaired sleep in chronic pain is gaining more scientific focus in recent years. Accumulating evidence suggests that maladaptive sleep hampers both neural and molecular control mechanisms (e.g., Haack et al., 2020; Sun et al., 2020). Our results lend additional support for the relation between impaired neural connectivity and sleep disturbance, and in addition, highlight the role of affective attention in pain chronicity.

Furthermore, our findings suggest that patients with chronic pain demonstrate a similar pattern of results to the one found in previous studies with anxious and sleep-deprived individuals (Simon et al., 2015; McTeague et al., 2018). However, these abnormalities correspond to different dimensions of symptoms: while in anxious individuals abnormal ssVEP pattern were correlated with affective symptoms, in our study ssVEP indices were correlated with somatic, but not depressive or anxiety-related disease manifestations. These results are resonating with the clinical application of the theory of constructed emotion (Barrett, 2017). According to this theory, emotional experiences are constructed based on core dimensions such as valance, arousal (together called affect), bodily perceptions (interoceptions), and their interaction with sensory and conceptual information. Accordingly, many disorders, including anxiety and chronic pain share core dysfunctions with respect to affective bias, by overweighting predictions related to negative affect (Barrett, 2017). At the same time, these disorders also present diverging manifestations concerning specific impairments. For example, in chronic pain, negative affect tendencies interact with ascending information from nociceptive and inflammatory pathways while in anxiety, the same negative affect tendencies may interact with social or counterfactual mentalization processes. Such conceptualization may assist in interpreting the findings that similar abnormalities in affect-biased attention correlate with different symptoms, as a function of the study population.

In this study, the relationship between the EEG indices and clinical symptom severity is correlational and not causal. The issue of whether the maladaptive attentional patterns of FMS patients predispose these individuals to develop chronic pain or vice versa is an issue that should be addressed using longitudinal studies. When such a longitudinal approach was implemented by Hashmi et al. (2013), they discovered that activation of emotion-related brain circuitry during pain perception predicted the development of later chronic pain. However, we are not aware of studies that have examined the relevance of neural signals during emotional processing (without the presence of pain) in predicting the development of chronic pain. We believe that such investigations can help to better understand the mechanisms of chronic pain. An additional limitation of this study is the lack of symptom severity assessment in the healthy control group. Such an assessment would have been of interest, as it would have allowed us to assess whether the relations between EEG indices and symptoms severity differ between FMS and HC. New transdiagnostic approaches that aim to use behavioral tasks to evaluate risk or responsiveness to treatment often assume that individual differences in task performance are predictive of symptom severity, also in sub-clinical levels (Huys et al., 2016). Additional measures regarding the symptoms severity of the HC could help to interpret the findings of the current study. In addition, a larger sample size would have allowed us to evaluate the predictive validity of the abnormal EEG indices in a subset of the sample. Such an approach would strengthen the internal validity of the findings presented in this study.

We used FMS as a model of a chronic pain disorder that heavily involves affective symptoms. The results presented pose the question of whether similar relations between biases in affective attention and chronic pain, will also be present in other pain conditions that do not involve such a burden of overt affective symptoms. Testing this issue in a new patient population, for example in individuals that suffer from ongoing, but not yet chronic pain, or in other clinical chronic pain conditions, would advance our understanding of the intricate relational mechanisms underlying pain-emotion associations as well as their temporal dynamics. Accordingly, such insights might help inform treatment decisions and improve diagnostics, as suggested here for FMS. In addition, the EEG-ssVEP methods that were used here allowed us to infer about attentional processes that are impaired in FMS and their relations to disease symptoms without relying on direct patient reports. However, this is a rather limited approach in terms of tracking brain network dynamics and additional insights might be gained from using fMRI and a similar behavioral paradigm. An additional approach that might of use in this respect could involve the implementation of psychophysical tests that correspond to specific neurobiological mechanisms such as conditioned pain modulation (CPM; Nir and Yarnitsky, 2015) or quantitative sensory testing (QST; Yarnitsky, 1997). Implementation of such tests, in addition to a long-term clinical trajectory evaluation, would potentially strengthen and verify the external validity of the findings presented in the current study. Lastly, these insights regarding the intimate relations between chronic pain and affective pathology is crucial in mitigating the clinical reports of chronic pain patients and affirm their experience regarding the all-encompassing effects of chronic pain on their lives by grounding this in better neurobiological understanding.

The results of this study emphasize the intimate involvement of affective attention, a key process of emotional construction, in chronic pain, and highlight the potential importance of quantitatively probing such processes when evaluating and treating populations suffering from complex somatic symptomatology. Moreover, these results may suggest that negative attentional bias is a core impairment in several disorders that has differential clinical manifestations stemming from additional impairments in other domains.

## Data Availability Statement

The raw data supporting the conclusions of this article will be made available by the authors, without undue reservation.

## Ethics Statement

This study was reviewed and approved by Tel-Aviv Sourasky Medical Center institutional review board. The patients/participants provided their written informed consent to participate in this study.

## Author Contributions

NG, AK, EB, TH, and HS: conception and design. NG, YZ, LW, and AG: data collection. NG, NP, and AK: statistical analysis. JA and HS: clinical evaluation and screening producer. NG: drafting of the manuscript. AK, JA, EB, TH, and HS: reading, editing, and conceptualizing the final version of the manuscript. All authors contributed to the article and approved the submitted version.

## Conflict of Interest

The authors declare that the research was conducted in the absence of any commercial or financial relationships that could be construed as a potential conflict of interest.

## Publisher’s Note

All claims expressed in this article are solely those of the authors and do not necessarily represent those of their affiliated organizations, or those of the publisher, the editors and the reviewers. Any product that may be evaluated in this article, or claim that may be made by its manufacturer, is not guaranteed or endorsed by the publisher.

## Abbreviations

ACR: American College of Rheumatology
BDI: Beck’s Depression Inventory
CI: confidence interval
FDR: False Discovery Rate
FMS: Fibromyalgia Syndrome
iPLI: inter-site phase-locking index
PSQI: Pittsburgh sleep quality index
ssVEP: steady-state visual evoked potentials
STAI-T: Trait Anxiety Inventory.
