# Supplementary material for: Abnormal Visual Evoked Responses to Emotional Cues Correspond to Diagnosis and Disease Severity in Fibromyalgia
Source: Front Behav Neurosci. 2022 May 4;16:852133. doi: 10.3389/fnbeh.2022.852133 (PMC9116473; doi:10.3389/fnbeh.2022.852133)
Supplement: Supplementary file 1 [file Data_Sheet_1.pdf]

Abnormal Visual Evoked Responses to Emotional Cues Correspond to Diagnosis and Disease Severity in Fibromyalgia

Supplemental Information

Stimulus and procedure

Each trial began with a 1 s presentation of an IAPS (International Affective Picture System) image with individual pixels scrambled to avoid contamination of the ssVEP with transient responses to the luminance gradient created by stimulus onset. Next, a total of 150 yellow dots (each 0.3°* 0.3° of visual angle) were superimposed on the scrambled image for 2917 ms. The scrambled background picture was then replaced by a neutral, or negative image that remained on the screen for the remaining duration of the trial (5834 ms). Dots were distributed randomly across pictures and flickered at a rate of 7.5 Hz while remaining inside the circle (6.9° visual angle) at all times. The yellow dots were "on" for six frames and "off" for eight frames. All dots remained in continuous motion throughout the trial, and each dot changed its position by 0.04° in a random direction with every ssVEP cycle (i.e., 7.5 times/s).

In a random subset of 50% of the trials, all dots moved coherently in the same direction (target), and participants were instructed to respond to coherent motion events with a mouse click, as quickly and as accurately as possible. Coherent motion of the targets occurred in one of four diagonal directions (45°, 135°, 225°, and 315°) at random. To produce a difficult and demanding perceptual detection task, coherent motion lasted for only four successive cycles of 7.5 Hz (i.e., 533.33 ms). Targets occurred unpredictably once (in half of the trials, in 43 of 90) or twice (in 3 of the 90 trials), with the remaining

trials consisting of random movement of the dots. The first possible coherent motion event was at 1170 ms after stimulus onset and the last coherent motion event was at 7000 ms. Only single-target or no-target trials occurring during picture presentation were included in the final analysis. Each trial lasted for 9751 ms, with interstimulus intervals varying randomly between 3000 and 5000 ms. Fixation was facilitated by presenting a white fixation dot at the center of the screen. Before the experiment, all participants performed 15 practice trials which included different distractors than the main experiment to familiarize themselves with task instructions.

Stimuli were presented on an LED monitor, set at a resolution of 1024*768 with a refresh rate of 60 frames/s (i.e., 16.66 ms refresh interval). In accordance with previous ssVEP work (Müller et al., 2008), we compared the effects of different background picture categories on the dot-evoked ssVEP: the neutral category was composed of pictures presenting people at work or cows while stimulus related to the negative category contained mutilated human bodies or snakes. A Positive distractor category was composed of pictures showing erotic couples or kittens. Each valence category included 30 pictures totaling to 90 pictures across the three categories (negative, positive and neutral). In our main statistical analysis, we compared between negative and neutral categories but not the positive category, as we did not have a specific hypothesis regarding the impact of positive valence and wished to avoid unnecessary statistical comparisons that would increase type I errors. Pictures were selected from the International Affective Picture System (IAPS, (Lang et al., 1997), a database designed to provide a standardized set of pictures for studying emotion and attention. For all IAPS images, valence and arousal ratings for neutral images were 5.34 and 3.26, for positive images were 7.04 and 5.58, and for negative images were 2.84 and 6.39,

respectively. All picture stimuli were grayscale pictures and were controlled for visual complexity (measured as .jpeg size) and matched for luminance using scripts from the MATLAB image processing toolbox. Picture stimuli were circular in nature and were cropped and adjusted such that the defining element of each picture was positioned at the center of a circle.

EEG pre-processing

EEG data were low-pass filtered offline at a frequency (3 dB point) of 40 Hz (12th order Butterworth filter with 24 dB/octave roll-off implemented in MATLAB). Single epochs of 9200 ms in length (400 ms before and 8800 ms after dot onset) were then extracted from the continuous EEG signal. Segments that contained amplitudes greater than 1 standard deviation above the median amplitude were excluded from further analyses.

Separation to an early and late time window

The separation into two time windows was based on previous investigations that were done using a similar paradigm and aimed to test the dynamics of affective attention as probed by ssVEP (Deweese et al., 2014; Müller et al., 2008). In these investigations, time window separation was done based on a visual inspection of the data. In the current study, we used a similar conceptual approach but added finer calibration of the time window definition that was based directly on the results of Ben Simon et al., 2015. Our definition is based on the time window in which significant differences in ssVEP were observed between neutral and negative distractors. We treated this as an early time window (0-900 ms after background stimulus onset). The late time window was defined

as any time later than 900ms, that was still in a part of the "target window" (originally defined at Deweese et al., 2014).

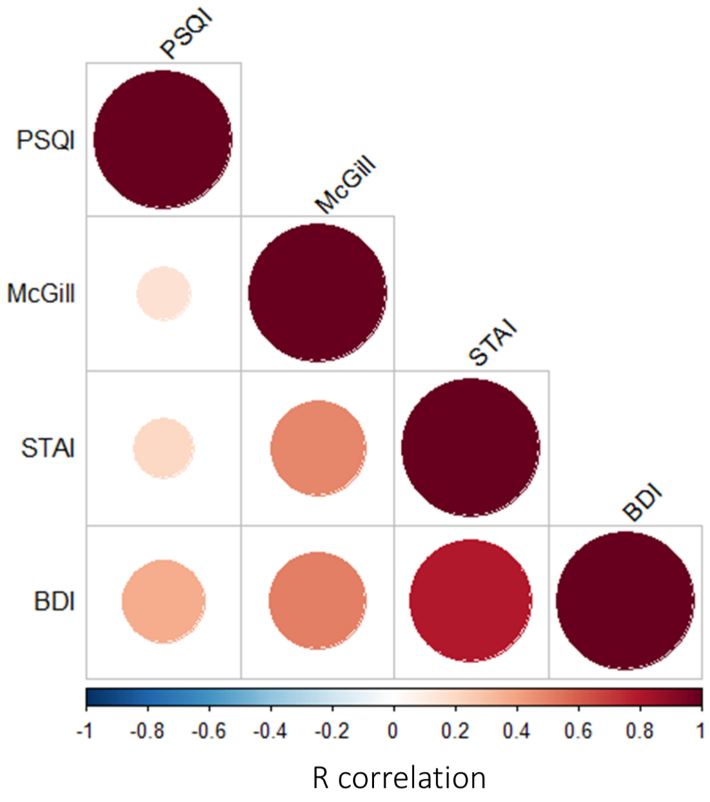

Supplementary Figure 1. **Correlation matrix of the interrelationship between clinical domains of FMS**. Size and color intensity are proportional to the correlation coefficients. PSQI: Pittsburgh Sleep Quality Index, McGill: pain questionnaire, BDI: Beck's Depression Inventory, STAI: Trait Anxiety Inventory.

References

Deweese, M. M., Bradley, M. M., Lang, P. J., Andersen, S. K., Müller, M. M., & Keil, A. (2014). Snake fearfulness is associated with sustained competitive biases to visual snake features: Hypervigilance without avoidance. *Psychiatry Research*, *219*(2), 329–335.

Lang, P. J., Bradley, M. M., & Cuthbert, B. N. (1997). International affective picture system (IAPS): Technical manual and affective ratings. *NIMH Center for the Study of Emotion and Attention*, *1*, 39–58.

Müller, M. M., Andersen, S. K., & Keil, A. (2008). Time course of competition for visual processing resources between emotional pictures and foreground task. *Cerebral Cortex*, *18*(8), 1892–1899.

Simon, E. B., Oren, N., Sharon, H., Kirschner, A., Goldway, N., Okon-Singer, H., Tauman, R., Deweese, M. M., Keil, A., & Hendler, T. (2015). Losing neutrality: The neural basis of impaired emotional control without sleep. *Journal of Neuroscience*, *35*(38), 13194–13205.
